# The Hospital World

**Published:** 1910-08-13

**Authors:** 


### The Hospital World.

### Personalia.

At the meeting of the House Committee of the Hull Victoria Hospital for Sick Children, Park Street, in the Board-room of the institution recently, the Chairman (Mr. G. F. Grant), on behalf of the Committee, presented a silver snuff-box, suitably inscribed, to Mr. Arthur G. Hill, member of the Committee and hon. treasurer of the institution, as a slight token of the esteem in which Mr. Hill is held by his colleagues on the Committee, and of their high appreciation of his indefatigable exertions for over a quarter of a century in the service and interests of the charity. In a few eloquent and appropriate words, Mr. Grant (whose own membership of the House Committee and of the Board of Management dates back to the 'eighties of the last century) feelingly alluded to Mr. Hill's long connection with the hospital and his valuable work on its behalf, and mentioned that he was elected by the members on the governing body in March 1885, hon. assistant treasurer in June 1889, and hon. treasurer, in succession to Mr. Francis R. Pease (resigned), in June 1907. Mr. Hill expressed his thanks for this concrete expression of his colleagues' esteem, and added that the work of the institution had always been a pleasure to him, especially when friends came forward so spontaneously at crucial moments to help him in its temporary pecuniary difficulties.

The League of Mercy.—His Serene Highness Prince Alexander of Teck, Grand President, and her Royal Highness Princess Alexander of Teck, Lady Grand President, presented the Order of Mercy to the under-mentioned ladies and gentlemen at St. James's Palace on Tuesday, July 26 :—Presidents : Lieut.-Col. Sir Adelbert Talbot, K.C.I.E. (Epsom-Esher), Dr. Scott (Islington, W.), Mr. Carl Hentschel (Strand), Mr. Carl Stettauer (Bermondsey). Lady Presidents : The Lady Battersea (Battersea), Lady Dimsdale (S. Paddington), Mrs. John Hamilton Evans (Horsham). Vice-President : Sir William Robert Clayton (Bucks). Lady Vice-Presidents : The Viscountess Dillon (Oxon), The Viscountess Esher (Berks), The Lady Garvagh (St. George's), The Hon. Mrs. Charles Lawrence (N. St. Pancras), The Hon. Mrs. Richard Moreton (Hants), Lady Clayton (Bucks), Lady Portal (Hants), Miss E. Bisdee (N. Kensington), Mrs. Charles Crawshay (Mid Essex), Mrs. Gardner (Romford), Miss Goddard (Brixton), Mrs. Hamilton (Deptford, Brockley), Mrs. Lee, of Hartwell (Bucks), Mrs. Sutton Sharpe (S. Paddington), Mrs. Bruce Shipway (Kingston-Surbiton), Mrs. Tyser (Berks), Mrs. Alfred Waley (Reigate), Miss Yolland (S. Paddington). Members : Dr. Chearnley Smith (Epsom-Esher), Mr. A. Campbell (St. George's), Mr. Bernard Kauffmann (E. Marylebone), Mr. Matthews (S. Kensington), Mr. J. Pike (S. Kensington), Mr. R. Silvester (Chelsea), Mr. H. G. Sneath (City), Miss Butler (Fulham), Miss Church (S. Kensington), Miss Edythe Enthoven (S. Paddington), Mrs. Freyberger (W. St. Pancras), Mrs. Godman (Fulham), Mrs. Groner (Hampstead), Mrs. Miller Hallett (Sevenoaks), Miss Lucy Hewitt (Epsom-Esher), Mrs. Howden (Islington West), Mrs. Lott (Fulham), Miss A. Pardoe (Eastbourne), Miss E. E. Stovell (Hampstead), Miss Kitty Wade (Sevenoaks), Miss D. Warden (Hammersmith), Miss Alice Waymark (Tonbridge), Miss E. Wynter (Westminster).

